# Barriers to accessing health care of older Chinese immigrants in Canada: a scoping review

**DOI:** 10.3389/fpubh.2024.1445964

**Published:** 2024-11-05

**Authors:** Change Zhu, Baoxiang Song, Christine A. Walsh, Prince Chiagozie Ekoh, Xuebin Qiao, Aijun Xu

**Affiliations:** ^1^School of Health Economics and Management, Nanjing University of Chinese Medicine, Nanjing, China; ^2^Jiangsu Research Center for Major Health Risk Management and TCM Control Policy, Nanjing University of Chinese Medicine, Nanjing, China; ^3^Faculty of Social Work, University of Calgary, Calgary, AB, Canada

**Keywords:** barriers, access, health care, older Chinese immigrants, Canada, scoping review

## Abstract

**Background:**

This scoping review aims to examine the extant literature and summarize findings related to barriers to accessing health care faced by older Chinese immigrants in Canada.

**Methods:**

We conducted a search of electronic databases for peer-reviewed articles using a comprehensive set of keywords without limiting the search to a specific time period. To be included in our review, articles had to meet the following criteria: (a) published in a peer-reviewed journal, (b) written in English, (c) provide a clear description of the methods used, and (d) respond to our research question, which focuses on identifying barriers to accessing healthcare for older Chinese immigrants living in Canada.

**Results:**

Fifteen papers were selected based on the criteria, and five main barriers were identified, which are ranked in descending order according to the number of times they were mentioned: culture and health beliefs (*N* = 13), language and communication (*N* = 7), structural and circumstances (*N* = 2), health literacy and information (*N* = 2), and demographic, social, and economic factors (*N* = 2).

**Conclusions:**

The issue of accessing healthcare for older Chinese immigrants in Canada is complex, as it involves multiple aspects that are relevant to both patients and healthcare providers. Our research findings suggest that the culturally and linguistically sensitive education programs, inter-sectoral coordination, and social support should be improved for older Chinese immigrants and those of other ethnic backgrounds.

## 1 Introduction

The demographic landscape across the globe has been shaped by a notable trend of international migration (1). Canada, as a popular destination for immigrants (2), has seen a substantial influx, with Statistics Canada (2021) reporting over 8.36 million immigrants by 2021, accounting for 23% of the overall population (3). This growing trend includes a significant number of immigrants primarily from Asia and the Middle East (4).

In urban centers, such as the Toronto Census Metropolitan Area, immigrants not only make up a considerable portion of the general population but are also a substantial part of the older adult demographic. For instance, while immigrants constitute 48% of Toronto's population, they represent 70% of those aged 65 and above (5). This pattern is reflected nationwide where, as of 2015, 30% of Canadians aged 65 or above were immigrants (6), highlighting the growing presence of older adult immigrants in the healthcare system.

Zooming in further, within Canada's visible minority population, Chinese residents form the second largest group, totaling 1.3 million from mainland China, Hong Kong, and Macao (5). Remarkably, 96% of older Chinese individuals (aged 60 and above) in Canada are immigrants (5), reflecting historical immigration patterns (7), family reunification (8), and economic opportunities (9). Given their significant representation, it is crucial to explore the specific healthcare access barriers faced by older Chinese immigrants, as they encounter distinct challenges not solely due to their population size but also due to cultural and linguistic differences. Unlike many other immigrant groups, older Chinese immigrants often face language barriers, unfamiliarity with Western healthcare practices, and traditional health beliefs that may conflict with mainstream medical approaches. These factors create unique barriers in accessing and utilizing healthcare services. Thus, targeted research is needed to better understand and address the specific healthcare needs of this population, ensuring more equitable access within the aging demographic.

The challenges faced by the aging immigrant population are profound and multifaceted (10). They not only grapple with the normal decline in physical functioning but also with acculturative stress, lifestyle changes, and the nuances of Western healthcare systems. These challenges are compounded for older immigrants, who experience more significant health issues compared to their non-immigrant counterparts. For example, older immigrants are more likely to report mental illnesses (11) and show a more pronounced decline in emotional functioning over time compared to non-immigrant older adults (12). This heightened emotional functioning vulnerability can be attributed to factors such as cultural adaptation stress and social isolation, which are less prevalent among non-immigrant older adults (13). As a result, immigrants may experience a sharper decline in emotional functioning over time. Furthermore, long-term immigrants (residing in Canada for over 10 years) also report deteriorating health conditions, indicating a gradual decline in immigrant health status (14, 15).

Despite Canada's publicly funded healthcare system designed to provide equitable access irrespective of age and immigration status, research indicates that older immigrants utilize healthcare services less frequently than younger ones, with data over two decades old still suggesting low utilization among this group (16).

Furthermore, existing studies have identified multiple barriers to healthcare access for older immigrants, including language and communication difficulties (17), transportation issues (18), and financial constraints (19). However, no study has specifically mapped out the barriers faced by older Chinese immigrants in accessing healthcare services in Canada. Although some of these challenges may overlap with those of other immigrant groups, the distinct cultural and linguistic barriers faced by Chinese older adults, coupled with traditional health beliefs, warrant a dedicated scoping review. Understanding these barriers is crucial for guiding future research aimed at improving healthcare access for older adults.

Hence, this scoping review aims to comprehensively examine the existing literature on the barriers older Chinese immigrants face in accessing healthcare services in Canada. Through this analysis, we seek to identify knowledge gaps and suggest directions for future research.

## 2 Methods

Arksey and O'Malley's five stages of scoping review framework was adopted for this review (20). The framework comprises five stages: identifying the research question, identifying the relevant studies, selecting studies, charting the data, and collating, summarizing, and reporting the results, each of which is described in the following section.

### 2.1 Stage 1: identifying the research question

To conduct a successful scoping review, it is essential to formulate focused research questions while keeping the investigation inclusive enough to avoid disregarding any valuable literatures (20). The review was guided by the research questions: (1) What original research has been conducted on the barriers to healthcare access faced by older Chinese immigrants in Canada? This served as the primary research question. (2) How can access to healthcare be improved for older Chinese immigrants in Canada? This functioned as the secondary research question.

### 2.2 Stage 2: identifying relevant studies

Firstly, expert consultation was sought to determine the most appropriate databases for the search. Following the recommendations of the expert, the following eight databases were systematically searched to identify relevant studies for review: Abstracts in Social Gerontology, PubMed, Social Services Abstracts, CINAHL, Social Work Abstracts, Sociological Abstracts, ScienceDirect, and Web of Science. Finally, the EndNote software was employed to organize the gathered literature.

### 2.3 Stage 3: selecting studies

**Figure 1:**
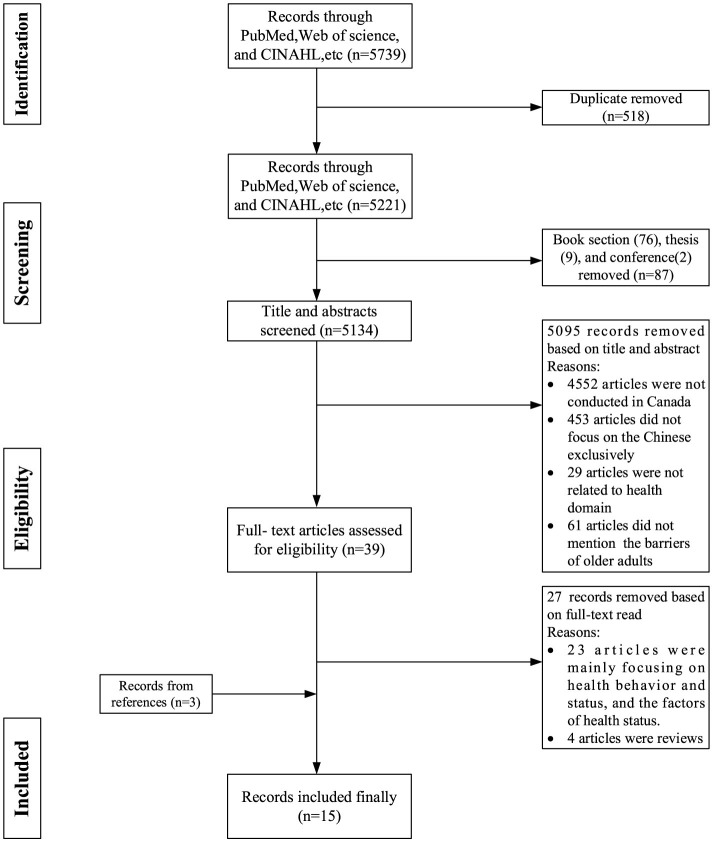
PRISMA flow diagram on the outcomes of the search strategies.

Our study exclusively considered research studies published in the English language. A total of 5,739 titles and abstracts from various sources, including PubMed (*N* = 2,958), CINAHL (*N* = 97), Social Work Abstracts (*N* = 5), Abstracts in Social Gerontology (*N* = 37), Social Services Abstracts (*N* = 1,100), Sociological Abstracts (*N* = 932), ScienceDirect (*N* = 331), and Web of Science (*N* = 279), were obtained during the initial literature search. The systematic identification and removal of duplicate references were conducted using EndNote software. Following the removal of duplicates (*N* = 518), book section (*N* = 76), thesis (*N* = 9), and conference proceedings (*N* = 2), a thorough review was conducted on the remaining 5,134 titles and abstracts using the established inclusion and exclusion criteria. Then, a two-step screening process was performed by two separate reviewers (ZC and PE). The initial step involved evaluating the titles and abstracts according to specified inclusion and exclusion criteria (see Table 1). To ensure a focused examination, only articles specifically studying older Chinese immigrants were selected for inclusion. Studies involving older individuals from other countries or ethnicities were excluded to maintain the specificity of the research population. This process resulted in the retrieval and evaluation of 39 full-text articles. At this step, 27 articles were excluded from the analysis as they focused on health behavior, health status, or the factors affecting health instead of the barriers to accessing health care. Also, despite the exclusion of related review papers, a manual search was conducted to identify relevant articles included within those reviews. As a result, 3 additional articles were included in the analysis. In total, 15 articles comprised the final sample for further analysis. The papers selected from the first step were then thoroughly reviewed in the second step. Any disagreements between the two reviewers were reconciled through consensus or by seeking a decision from a third reviewer (CW) to determine the final papers to be included. Since no existing guidelines exist for reporting scoping reviews, the flow of articles included in this review was documented using the Preferred Reporting Items for Systematic Reviews and Meta-Analysis (21) (see Figure 1).

**Table 1:** Inclusion and exclusion criteria for study selection.

**Inclusion criteria**	**Exclusion criteria**
The study must be published in a peer-reviewed journal	Gray literature, published books, and conference papers
The study must be written in English	Published papers in languages other than English
The study must have a clear description of the methods used	Other relevant non-empirical papers
The subjects must be older Chinese immigrants in Canada	Older Chinese immigrants in other countries and young Chinese immigrants
The study must be relevant to the research question, for instance, focus on the barriers to access to health care	Papers not focusing on barriers to access to health care for older Chinese immigrants

### 2.4 Stage 4: charting the data

Essential information from the selected articles was collected and organized into a data chart format. This chart included details such as the first author, year of publication, study sites, aims, methods, mentioned barriers and interventions. Two evaluators (ZC and PE) separately evaluated the initial version of the data chart form using five randomly selected papers. Following mutual discussions and feedback, the chart form was modified. The updated chart form was then thoroughly reviewed and accepted by the team (ZC, PE, and CW) and was utilized to gather information from all the studies included in a narrative form.

### 2.5 Stage 5: collating, summarizing, and reporting the results

The main objective of scoping studies is not to assess the literature's quality but to create a comprehensive overview of existing research or establish a thematic framework that offers a broad understanding of the research conducted in a specific area of inquiry. The data were analyzed using a thematic approach (22). In the data synthesis phase, NVivo software was employed to facilitate the thematic analysis. The imported literature underwent an initial review, with key concepts and themes marked using the software's tagging and annotation features. A preliminary coding framework was established, creating nodes within NVivo to represent different themes or concepts identified in the literature. Systematic coding was then applied to each piece of literature, associating relevant text with the established nodes. Utilizing NVivo's analytical tools, the coded data was synthesized to identify recurring themes across multiple pieces of literature. The coding process underwent validation and cross-checking within NVivo to ensure consistency and accuracy, involving collaborative efforts and discussions among team members. To further guarantee consistency and validate the fifth stage of the process, two reviewers (ZC and PE) each conducted their own independent thematic analysis of the literature. The research team collectively approved the final results after critical evaluations and discussions.

## 3 Results

### 3.1 Study characteristics

Out of the 15 studies, most were quantitative (*N* = 9, 60%); the remainder were qualitative (*N* = 3, 20%); and mixed-methods (*N* = 3, 20%), as shown in Tables 2 and 3. Concerning the research location, nine studies were conducted in Ontario, primarily in Toronto; five studies were conducted in Alberta, mainly in Calgary and Edmonton and in addition, six studies were conducted in British Columbia, particularly in Vancouver and Victoria. Further, four studies were conducted throughout Canada without specifying a particular region or city. In addition, Lai, Tieu, and Todd were the primary authors dedicated to research on barriers to accessing health services for older immigrants in Canada. A total of 9 papers were completed by these authors, with Lai, the lead author contributing five papers and Tieu and Todd each leading two papers, respectively. Among the selected papers, 12 out of 15 were conducted a decade ago, while only three papers were conducted within the past 10 years, indicating a dearth of recent studies addressing this particular topic.

**Table 2:** Study description and mentioned barriers.

**References**	**Study site(s)**	**Aims**	**Sample Size**	**Methods**	**Key findings (barriers mentioned)**	**Interventions**
Chang et al. (23)	Canada (cities and provinces not stated)	To explore how Mandarin- and English-speaking Chinese women experience Pap testing	13 older Chinese women	Method: qualitative Analysis: thematic content analyses	Preference for Chinese medicine, culturally bound perceptions of sex and sexuality, and differences in the institutionalization of medical care between country of origin and country of residency.	Efforts could be made to align the significance of Pap testing with Chinese medicine philosophies. Educating Chinese women about the etiology of gynecological diseases and dispelling the erroneous belief that sexual promiscuity is a cause. Additionally, the approach to conducting Pap testing may need to be reconsidered.
Lai and Chau (24)	British Columbia: Victoria and Vancouver; Alberta: Calgary and Edmonton; Manitoba: Winnipeg; Quebec: Montreal; Ontario: Toronto	To explore the barriers that older Chinese immigrants face when accessing healthcare services in Canada	2,214 older Chinese	Method: quantitative Design: cross-sectional Analysis: logistic regression	Service barriers were related to administrative problems in delivery, cultural incompatibility, personal attitudes, and circumstantial challenges. Specifically, barriers were identified as being female, single, lack of transportation, a shorter length of residency in Canada, low income, lack of social networks, and “traditional” health beliefs.	Social work interventions should strengthen support and resources for the vulnerable groups identified in the findings. Service providers should adjust service delivery to better serve older immigrant adults who still maintain strong Chinese cultural values and beliefs.
Lai and Chau (25)	British Columbia: Victoria and Vancouver; Alberta: Calgary and Edmonton; Manitoba: Winnipeg; Quebec: Montreal; Ontario: Toronto	To examine the effects of service barriers on the health status of older Chinese immigrants in Canada	2,214 older Chinese	Method: quantitative Analysis: descriptive statistics and multiple regression analysis	Service barriers are related to administrative problems, personal attitudes, and circumstantial difficulties. Also, the service barriers in the areas of ethnic, language, or cultural differences between the service providers and the older Chinese were mentioned.	Sociocultural context, culturally appropriate psychosocial education programs, appropriate transportation arrangements should be considered.
Lai and Chappell (26)	British Columbia: Victoria and Vancouver; Alberta: Calgary and Edmonton; Manitoba: Winnipeg; Quebec: Montreal; Ontario: Toronto	To understand the prevalence and predictors of Traditional Chinese Medicine (TCM) use by older Chinese immigrants in Canada	2,167 older Chinese immigrants	Method: quantitative Design: cross-sectional Analysis: logistic regression	Country of origin, Chinese health beliefs, social support, city of residency, and health variables were the common predictors of using a form of TCM. Culture-related variables are important in determining use of TCM.	Physicians caring for older Chinese immigrants should consider the potential use of herbs and herbal formulas alongside Western medicine. Cultural factors should be taken into account when delivering treatment and health services. Understanding patients' health beliefs is crucial, as it often influences their choice of health services. Thus, inquiring about patients' health beliefs associated with their concerns becomes important.
Lai and Kalyniak (27)	British Columbia: Victoria and Vancouver; Alberta: Calgary and Edmonton; Manitoba: Winnipeg; Quebec: Montreal; Ontario: Toronto	To identify predictors of use of annual physical examination by aging Chinese Canadians	2,272 older Chinese Canadians	Method: quantitative Design: cross-sectional Analysis: hierarchical logistic regression	Primary affecting factors of access to health care are as follows, marital status, gender, length of residency in Canada, Chinese ethnic identity, social support, number of illnesses, dependency in instrumental activities of daily living (IADL), and depressive symptoms.	Strengthened ethnic identity may serve to enhance one's social support network, which in turn facilitates the use of annual physical examinations.
Lai (28)	British Columbia: Victoria and Vancouver; Alberta: Calgary and Edmonton; Manitoba: Winnipeg; Quebec: Montreal; Ontario: Toronto	This study examines the effect of cultural factors on the depressive symptoms reported by older Chinese immigrants in Canada	1,537 older Chinese	Method: quantitative Design: cross-sectional Analysis: hierarchical logistic regression	Cultural differences are a key barrier to accessing health care.	The healthcare delivery system needs to better accommodate and be attuned to the distinct ethnic and cultural variations among older immigrants.
Jackson et al. (29)	British Columbia	To examine barriers and facilitators of screening mammography among Chinese Canadian women	213 older Chinese women	Method: quantitative Design: cross-sectional Analysis: bivariate analyses with chi square tests; unconditional logistic regression models	Chinese traditional culture, such as faith in God or Buddha, was a barrier to mammograph screening.	Interventions involving education provided by both medical professionals and family members should be considered.
Kong et al. (30)	Canada (provinces or cities not stated)	The objective of this study was to assess attitudes toward the use of TCM for COVID-19 among Chinese immigrants in Canada during the early stage of the COVID-19 pandemic.	754 Chinese participants, including 216 older Chinese	Method: quantitative Design: cross-sectional study Analysis: descriptive analysis and multiple logistic regression	Attitude toward TCM affects access to health care among Chinese immigrants. Older Chinese immigrants were more confident in the effectiveness of TCM for preventing and managing the symptoms of COVID-19.	Further cultivate trust in traditional Chinese medicine among Chinese immigrants in Canada.
Liu and McDaniel (31)	Ontario	To examine the service gaps, needs, and barriers of Chinese Ontarians living with heart disease and stroke	26 senior Chinese	Method: qualitative Analysis: narrative research	Identified barriers are as follows, insufficient home care hours and respite programs for caregivers, lack of interpretive services in emergency rooms, and lack of Mandarin-speaking family doctors and specialists as well as culturally appropriate long-term care services for ethnic minority seniors.	Hire more Chinese-speaking healthcare professionals, increase public investment in culturally sensitive health services for ethnic minority seniors, and establish a well-integrated care system to enhance effectiveness and accessibility for caregivers and survivors of visible minority groups in Canada.
Matthew et al. (32)	Greater Toronto Area (GTA)	To understand the experience of widowhood amongst older Chinese immigrant women living in Toronto, Canada	20 older Chinese windows	Method: qualitative Design: cross-sectional Analysis: thematic analysis	Language and communication issues were commonly cited as barriers to receiving formal, public health services.	Accessing traditional medical services enables older Chinese Canadian widows to overcome language barriers in communication.
Tieu and Konnert (33)	Canada (provinces a and cities not stated)	Determining Chinese cultural beliefs how to predict attitudes toward mental health help seeking, to assess mental health utilization, and to assess intentions to utilize mental health services among older Chinese immigrants in Canada	149 older Chinese adults	Method: quantitative Analysis: hierarchical multiple regression, descriptive analysis, and *t-*tests	Older Chinese participants with strong Chinese cultural belief exhibited less positive attitudes to mental health seeking compared with those who has low Chinese cultural belief. Therefore, strong Chinese cultural belief was a barrier to accessing mental health care.	Greater perceived social support contributes to positive help-seeking attitudes.
Tieu et al. (34)	Canada (cities and provinces not stated)	To investigate depression literacy among older Chinese immigrants in Canada and compared their literacy to Canadian-born participants of the same age who were part of a larger population-based survey	53 older Chinese adults	Method: mixed Design: cross-sectional Analysis: a case vignette and multiple χ2 analyses	Lack of recognition cultural/linguistic barriers and Chinese beliefs affect the access to mental health services.	Self-help interventions (physical activity, getting out and about more, taking courses) should be implemented in a culturally sensitive manner.
Tjam and Hirdes (35)	Kitchener/Waterloo area in Ontario	The objective of this study is to explore health, psychosocial and cultural determinants of use of TCM and Western medicines among Chinese-Canadian older persons	106 older Chinese adults	Method: quantitative Design: cross-sectional study Analysis: multivariate logistic model	Health, psycho-social and cultural factors were significant determinants for medication use. The barriers to utilizing the western medical service were related to their preference for the use of TCM.	Educational programs are essential for both Chinese-Canadian older individuals and healthcare providers to grasp the proper utilization of Western and Traditional Chinese Medicine (TCM) treatments.
Todd et al. (36)	Ontario	Exploring predictors of breast and colon cancer screening for older Chinese immigrant women	103 older Chinese women	Method: mixed Design: cross-sectional Analysis: bivariate analyses, thematic analysis; logistic regression analysis; chi-square test; Fisher's exact test; t-test	Barriers identified for older Chinese women include a lack of health literacy surrounding screening practices, cultural factors (such as modesty), language/communication barriers, a lack of physician recommendation, and the gender of the physician.	Physician recommendation, having a female physician and high or moderate proficiency in English predicted current mammography screening.
Todd and Hoffman-Goetz (37)	Ontario	Exploring the preferences and behaviors of cancer information seeking of older Chinese immigrant women.	50 older Chinese women	Method: mixed Design: cross-sectional Analysis: thematic analysis; chi-square test; Fisher's exact test	Language and limited time with physicians were identified as barriers to accessing cancer information.	Cancer information that is in line with cultural norms and languages is needed.

**Table 3:** Number of studies listing specific barriers to accessing healthcare.

**Ranks**	**Barrier**	**N**	**References**
1	Cultural difference, health belief, and preferences for Chinese traditional medicine	13	(23–26, 28–36)
2	Language and communication barrier	7	(24, 25, 31, 32, 34, 36, 37)
3	Structural and circumstances	2	(23, 24)
4	Health literacy	2	(36, 37)
5	Demographic, social, and economic barriers	2	(24, 28)

### 3.2 Thematic categorization of access to healthcare barriers

Accessing healthcare services can be more challenging for older individuals who belong to ethnic minority groups due to the unique barriers they may face. Increasing globalization and migration have led to a growing number of older immigrants facing significant challenges in accessing health care, primarily due to factors such as cultural disparities, language barriers, and unfamiliarity with the local healthcare system (17). This is particularly true for older Chinese immigrants in Canada. We have classified the 15 articles into five main categories based on their focus areas and specific thematic categorization of the barriers to accessing healthcare by older Chinese immigrants in Canada as follows. To provide specific details, among the selected articles, 13 articles addressed barriers associated with cultural differences, health beliefs, and preferences for Chinese traditional medicine. Seven articles focused on language and communication barriers, while 2 articles examined barriers related to structural and circumstantial factors. Also, 2 articles discussed barriers related to health literacy. Lastly, 2 of them discussed barriers related to demographics, social and economic factors.

#### 3.2.1 Theme- I cultural and health beliefs barriers

Culture involves the distinctive ways of thinking and acting that set individuals or groups apart from one another (38). As people participate in social groups and share experiences, culture becomes deeply embedded in their identity, shaping their attitudes and behaviors (39). Their cultural tendencies influence the way people approach their health and make decisions related to healthcare. Thus, healthcare providers must have an understanding of their patient's cultural backgrounds in order to provide optimal care.

Cultural differences have been shown to affect the healthcare access of older Chinese immigrants in Canada (24). Over half of the articles identified cultural differences as a significant factor to access to health care among older Chinese immigrants in Canada (23–26, 28–36). For example, older Chinese immigrants may hold different beliefs and attitudes toward health and health care compared to the Canadian population. Also, immigrants originating from Hong Kong may exhibit distinct cultural values that differ from those of Canadian culture, thus resulting in a higher likelihood of reporting obstacles to accessing services compared to immigrants from other regions (24). Cultural incompatibility resulting from the unique and strong cultural values held by older Chinese immigrants, which differ from the Western perspective, significantly impacts their utilization of healthcare services (24, 25). Moreover, due to the modesty culture and emphasis on privacy in China, Chinese women tend to be conservative in sexual health, such as breast examination, thus exhibiting a shorter continuance of mammography use (36). Even some older Chinese females hold traditional beliefs, such that cancer can be prevented through faith in God or Buddha, thus presenting a low frequency of

mammography (29). Chinese women in Canada generally prefer the compulsory and depersonalized manner in which Pap tests are performed in China, as this can help to reduce the embarrassment associated with undergoing the test (23). Again, Chinese health beliefs and values are associated with mental health help-seeking attitudes among older Chinese immigrants in Canada. Strong adherence to Chinese health culture predicts a lower likelihood of positive attitudes toward seeking healthcare in Canada (33, 34). Hence, it was reported that older Chinese widows in Canada show a preference for services from private TCM practitioners, acupuncturists and herbalists because it is culturally relevant and easily understood (32). In addition, a strong health belief in the effectiveness of traditional Chinese medicine, deeply rooted in the Chinese mindset, has decreased the desire to access Western healthcare services in Canada (30).

#### 3.2.2 Theme-II language and communication barriers

The most common languages of Canadian Chinese immigrants are Mandarin and Cantonese (35). Therefore, a considerable number of Canadian Chinese immigrants may not be able to communicate effectively with doctors in Canada. To be more specific, 7 out of 15 studies mentioned language barriers to access to health care services (24, 25, 31, 32, 34, 36, 37). When seeking healthcare or accessing healthcare information, language fluency is a primary concern (37). A qualitative study by Martin-Mathews et al. reported that widows did not recognize the direction to the clinic and missed the appointment with a doctor as a result of limited English proficiency (32). Chinese older immigrants often also underutilize mental health services due to linguistic barriers (34). Furthermore, limited English proficiency resulted in underutilization of specific health services, such as colon cancer screening (36). Also, language and communication have been identified as significant barriers not only for older Chinese immigrants to access healthcare but also for healthcare providers who may be insensitive or unresponsive to their need (25). For instance, Lai and Chau found that most of the top service barriers were related to language among 2,214 older Chinese individuals, specifically manifested as the physicians not speaking their preferred language (24). Lack of Mandarin-speaking physicians and interpretive services are the main gaps in Canadian healthcare services, which have been shown to hinder older adults with heart disease and stroke access to healthcare (31).

#### 3.2.3 Theme-III structural and circumstances barriers

Besides languages and cultural barriers, structural and circumstances barriers were also found to pose hindrances to seeking healthcare by older Chinese immigrants. Structural and circumstantial barriers are a persistent and comprehensive concept that includes health system patterns, transportation, waiting list, geographical distance, health-related information availability, institutionalization variations, and other related factors. For instance, the absence of transportation was a significant obstacle to obtaining formal health care (24). A cross-sectional study on 2,214 older Chinese immigrants by Lai and Chau study revealed that insufficient transportation was a potential barrier to access to healthcare services (24). Furthermore, they also reported that administrative issues in service delivery, such as excessively long waiting lists, inconvenient office hours, and overly complex procedures, can reduce the motivation to seek health care services (24). A study has noted that older immigrants, in particular, are unwilling to wait for extended periods to access health services (24). Finally, Institutionalization variations in health care between China and Canada were found to be related to health utilization behavior, preference for Chinese institutionalization of medical care predicting a lower prevalence of pap screening (23).

#### 3.2.4 Theme-IV health literacy and information barriers

Health literacy and access to information play crucial roles in enabling immigrants to effectively utilize health services. Previous studies have demonstrated that the lack of health knowledge is a barrier for immigrants to access and utilize health services (40, 41). Furthermore, improved health literacy among immigrants has been shown to substantially enhance their utilization of primary healthcare services. For instance, older Chinese women in Canada with greater health literacy are more likely to participate in breast and colon cancer screenings compared to those with lesser knowledge (36). Additionally, research indicates that lower health literacy among older Chinese immigrant women, particularly those who are English as a Second Language (ESL) speakers, often results in a preference for interpersonal sources of health information, such as consultations with doctors or discussions with family members, over written materials. This reliance on interpersonal sources, while beneficial, may limit their exposure to comprehensive health information and reduce their engagement in preventive health behaviors, such as cancer screening (37). These insights emphasize the importance of targeted educational interventions to increase health literacy and facilitate better access to healthcare among immigrant populations.

#### 3.2.5 Theme-V demographic, social, and economic barriers

When studying barriers to healthcare utilization among older Chinese immigrants in Canada, Lai identified several barriers, which are as follows: female gender, being single, being an immigrant from Hong Kong, and shorter length of residency in Canada (28). To be more specific, the studies reported that gender exerts an effect on healthcare access as being female, to some extent, is a barrier to accessing the health care (24). Also, being single decreased the probability of utilization of health care (24). Furthermore, Lai and Chau (25) mentioned that discrimination based on ethnicity and age is likely the cause of the issues faced by older Chinese immigrants when trying to access healthcare services (24).

## 4 Discussion

### 4.1 Discussion on five primary barriers

Based on the thematic analysis conducted above, five primary barriers to accessing healthcare services among older Chinese immigrants in Canada have been identified, as shown in Table 3. These barriers include cultural differences, language and communication, structural and circumstantial factors, health literacy and information, as well as demographic, social, and economic status. Consequently, it is imperative to delve deeper into these barriers and engage in further discussions.

#### 4.1.1 Culture difference

Cultural barriers were discovered to be particularly noteworthy among the five types of barriers identified in this study. The challenge of limited cultural competency hindering access to healthcare is not unique to Canada. Other countries with high immigration rates, such as Australia and the United States, are also facing similar predicaments (42, 43). Therefore, it is imperative to pay attention to cultural variation between different ethnicities, as depicted in the Chinese and Canadian context found in this research. Given the diverse array of cultures represented in Canada, healthcare professionals must possess a decent comprehension of the subtleties associated with their patients' cultures in order to deliver culturally sensitive care. As a previous study indicated, Canadian physicians must receive culturally appropriate medical education (44). To be more specific, it would be beneficial to incorporate appropriate cultural competency training into the curricula of medical education programs (45). The training should consider the challenges immigrants face in Canada when seeking healthcare. Cultural programs should be tailored to older Chinese clients. This measure can increase physicians' understanding of this matter and improve their preparedness to deliver care.

#### 4.1.2 Language and communication

Among all the obstacles to healthcare for this population, communication difficulties have been extensively researched (46). Language and communication have been identified as significant barriers not only for older Chinese immigrants to access healthcare but also for healthcare providers (25). It was noted that healthcare providers may experience frustration when they are unable to comprehend their patients' requirements or concerns (47). Some healthcare facilities utilize professional translators to address language barriers, but healthcare providers may feel uncomfortable communicating through an interpreter due to uncertainty about the interpreter's appropriate role and the need for confidentiality (48). As is well known, communicating through a translator or other support person, who lacks proficiency in medical terminology, can be challenging in accurately conveying a patient's feelings to a physician (49). Usually, interpreters are instructed not to express their opinions or add their personal comments, which means that they should refrain from providing extensive “cultural interpretation” and instead leave this task to the patient and physician. Physicians then face the challenge of comprehending the diverse cultures of the patients they treat in their aim to deliver “culturally competent” care (50). Additionally, most provincial health policies do not typically cover professional translator services, leading to significant variation in availability throughout Canada (51). Although governments and healthcare service providers have attempted to address barriers to immigrant healthcare by providing brochures or pamphlets printed in different languages, relying solely on printed material may not be sufficient to make a significant impact.

#### 4.1.3 Structural and circumstances

The circumstantial obstacles were barriers associated with insufficient transportation, unfavorable weather conditions for travel, excessively lengthy waitlists, inconvenient office hours, and complex procedures. The above barriers severely hinder the desire to utilize health care for older immigrants. It is well acknowledged that many older adults are unable to navigate public transit (32). Therefore, lack of transportation will lead to less mobility (52), a commonly reported barrier to accessing service (53). Due to financial constraints, older immigrants often have to work multiple jobs, making their time particularly limited (51). Therefore, they are afraid to wait for extended periods to access healthcare services (54).

#### 4.1.4 Health literacy

As depicted above, the low rates of healthcare utilization among older Chinese immigrants might be attributed not only to their limited awareness of healthcare coverage and service availability but also to their insufficient comprehension of the significance and advantages of receiving health examinations (27). Ballantyne et al. found that older immigrants from China often avoided discussing their self-care and alternative healthcare practices with their physicians in their research on medication usage among a diverse group of older immigrants (55). It can be inferred that older Chinese immigrants lack awareness of health promotion and have low health literacy. Also, health literacy is significant for access to healthcare services, given the complexity of the health system and information (56). Therefore, diminishing the lack of health knowledge as a crucial barrier among immigrants could be achieved by enhancing their comprehension of the Canadian healthcare system through public sensitization programs.

#### 4.1.5 Demographic, social, and economic status

We also discovered that the socioeconomic status of older immigrants poses a hindrance to access to healthcare. Upon their arrival in Canada, older immigrants frequently encounter financial difficulties that result from limited employment prospects (57), given that they are mostly retired or generally beyond the typical age of retirement in Canada (32). Also, older immigrants with low socioeconomic status or those who have recently immigrated are especially at a disadvantage as they are frequently excluded from the Canadian pension system due to their limited duration of employment in Canada (58). The Healthcare Act of Canada, enacted in 1984, stipulates that all individuals in Canada, regardless of their immigrant status, are entitled to equal access to healthcare services (59). Although healthcare is accessible to everyone in Canada, immigrants often prioritize working multiple jobs to support their families or accept low-paying positions over seeking healthcare, even when they are unwell (51). In addition, the lack of health insurance is another significant barrier older Chinese immigrants face in accessing health care, such as optometry, dentistry, and therapies, which are considered to be extended health care and not covered by public health insurance (17).

### 4.2 Policy and implications

From the scoping review on barriers to healthcare access for older Chinese immigrants in Canada, five key obstacles were identified: cultural and health beliefs, language and communication, structural circumstances, health literacy and information, and demographic, social, and economic barriers. To address these challenges, recommendations for policy, practice, and research were put forward.

In terms of cultural and health beliefs barriers, healthcare providers should align their practices with traditional Chinese medicine (TCM) philosophies. For example, integrating TCM perspectives with western medical practices, such as reconsidering the approach to pap testing, can help bridge the cultural gap. Educating healthcare professionals on Chinese health beliefs and providing culturally sensitive care are essential steps. The establishment of multicultural healthcare teams that integrate TCM alongside western medicine will enhance the trust of older Chinese immigrants in the healthcare system. Physicians should also consider using TCM-based treatments alongside western interventions, especially in areas like herbal formulas, to respect patient preferences. Additionally, providing accessible cancer information in line with Chinese cultural norms will further enhance healthcare literacy and trust in preventive care services, as cancer prevention and treatment beliefs are often deeply tied to cultural perspectives. For language and communication barriers, language barriers can be mitigated by hiring more mandarin-speaking healthcare professionals and offering language training to current staff. The availability of multilingual healthcare materials is crucial for ensuring that older Chinese immigrants can understand the healthcare services available to them. Further, healthcare institutions should develop systems that enable clear communication in culturally sensitive ways, such as providing interpreter services in emergency rooms. These efforts will help overcome communication challenges and ensure that older Chinese immigrants have access to necessary healthcare services. Regarding structural and circumstances barriers (administrative and logistical), such as administrative issues and transportation challenges, they can be alleviated by improving inter-departmental coordination and developing seamless referral systems. Social work interventions should be adjusted to cater to the specific needs of older Chinese immigrants who face logistical challenges like limited access to transportation. Healthcare providers should offer transportation assistance and ensure that medical services are located in easily accessible areas. Furthermore, healthcare delivery systems must be more culturally attuned to address the administrative difficulties faced by older Chinese immigrants, especially those who have recently relocated or lack adequate social support networks. With respect to health literacy and information barriers, health literacy interventions should focus on developing culturally appropriate and easy-to-understand health information materials. Campaigns aimed at raising awareness about health literacy, especially concerning screening practices such as mammography, should be tailored to older Chinese immigrants. These campaigns should highlight the importance of preventive healthcare services and provide clear guidance on how to access them. Physician recommendations for screenings, combined with culturally relevant education programs, will help increase participation in preventive health services. Finally, to address demographic, social, and economic barriers, advocating for social justice policies that ensure equitable access to healthcare services for older Chinese immigrants is crucial. Tailored social services, such as enhancing social support networks and providing psychosocial education programs, are critical to improving access. Specific efforts should be made to support those with limited financial resources, ensuring that healthcare is affordable and accessible. Strengthening ethnic identity and community support networks can also facilitate better healthcare access by promoting annual physical exams and routine health check-ups. Lastly, research should continue to investigate the impact of social and economic inequalities on healthcare access for this vulnerable population, with the goal of informing future policy changes.

However, while some of the articles included in this review were published over 10 years ago, and 3 of them more than 20 years ago, the challenges identified—such as language barriers, cultural differences, and healthcare system navigation—are long-standing issues that may persist today. Further research is needed to assess whether these barriers continue to affect older Chinese immigrants' access to healthcare services and to determine the most appropriate interventions in the current context.

### 4.3 Strengths and limitations

Compared to studies focusing on the general older immigrants, this was the first scoping review to our knowledge to focus on mapping out and documenting the barriers to accessing health care for older Chinese immigrants in Canada. The identified five main barriers, such as culture and health beliefs, language and communication, health literacy and information, structural circumstances, and demographic, social, and economic, identified in the study give implications to the government to improve the health care system and provides better service to ethnic older immigrants. However, this article was restricted to several limitations. Firstly, some of the articles included in this review were published over 10 years ago, with 3 of them being more than 20 years old. Although the barriers identified remain relevant, the age of some studies may affect the direct applicability of the findings to the current context, highlighting the need for updated research in this area. In addition, our study focused exclusively on barriers to accessing healthcare for older Chinese immigrant Canadians and did not address obstacles faced by other ethnic immigrants or non-immigrants, such as refugees and temporary Chinese residents. Due to differences in the populations examined, the barriers identified in this research may not be universally applicable to all older Chinese immigrant groups.

## 5 Conclusions

Scoping reviews are particularly beneficial for subjects that have not been thoroughly reviewed previously (20). From the above analysis, our scoping review found that the majority of studies (*N* = 12) were conducted decades ago, which shows a gap in recent understanding of barriers to healthcare for older Chinese immigrants. Hence, this indicates a new for more recent studies to better understand contemporary challenges and barriers to accessing healthcare by older Chinese immigrants. A total of 15 papers were included and five primary barriers were identified, which are ranked in descending order of importance as follows: culture and health beliefs (*N* = 13), language and communication (*N* = 7), structural circumstances (*N* = 2), health literacy and information (*N* = 2), and demographic, social and economic (*N* = 2).

The findings highlight the urgency of addressing these barriers to improve the health and wellbeing of this population. Moving forward, it is crucial to consider the policy, practice, and research implications. Addressing the identified barriers has the potential not only to enhance the health outcomes of older Chinese immigrants but also to contribute significantly to reducing healthcare disparities and costs in the long run.

## Data Availability

The original contributions presented in the study are included in the article/supplementary material, further inquiries can be directed to the corresponding authors.
